# Supplementary material for: Diffusion Tensor Imaging Changes Do Not Affect Long-Term Neurodevelopment following Early Erythropoietin among Extremely Preterm Infants in the Preterm Erythropoietin Neuroprotection Trial
Source: Brain Sci. 2021 Oct 16;11(10):1360. doi: 10.3390/brainsci11101360 (PMC8533828; doi:10.3390/brainsci11101360)

Supplementary Materials:

Supplemental Figure S1. Location of white and grey matter ROIs. Panels A and B show in red the location of grey matter ROIs in the basal ganglia bilaterally (a), and an area of occipital grey matter (b). Panel c shows in yellow an area of white matter located in the cingulate region, and d shows the location of the occipital white matter.

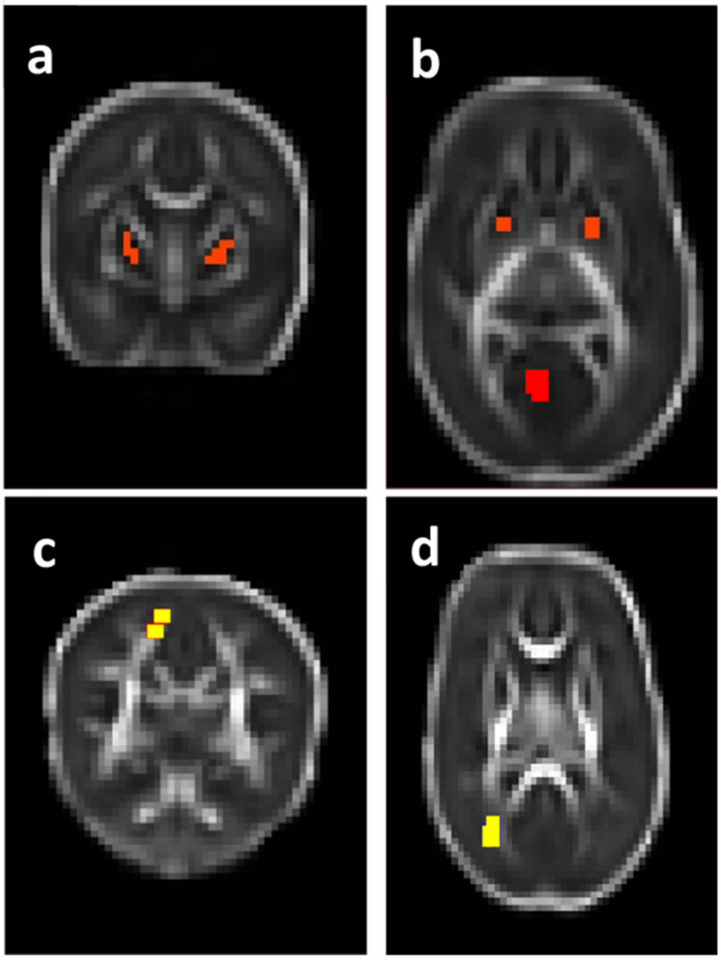

Supplemental Figure S2. Connectivity map. The nodes are brain regions in the JHU MNI SS WMPM atlas, and their coordinates, sizes, and colors represent the centroids, nodal strengths, and modules of the regions, respectively. The edges represent the connections between different brain regions (top 15% displayed). The structural connectivity strengths are presented as the radius of the edges, and long-distance connections (>90 mm) are colored in orange.

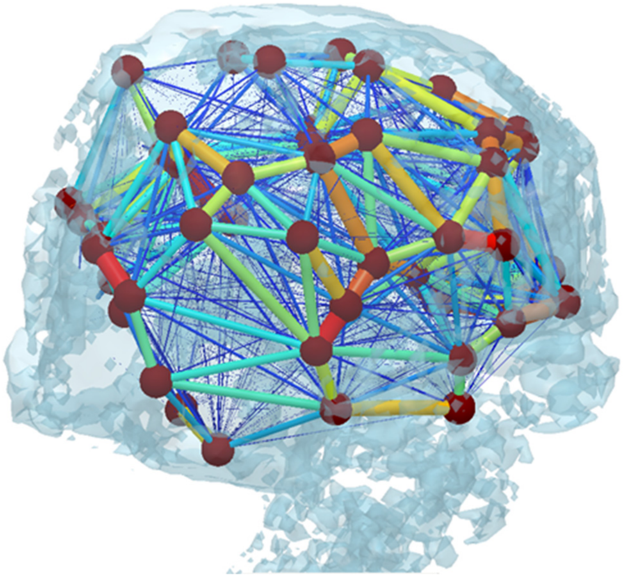

Supplement: Supplementary file 1 [file brainsci-11-01360-s001.zip › brainsci-1410518-supplementary.pdf]
